# Effects of Constant Flow vs. Constant Pressure Perfusion on Fluid Filtration in Severe Hypothermic Isolated Blood-Perfused Rat Lungs

**DOI:** 10.3389/fmed.2016.00070

**Published:** 2016-12-23

**Authors:** Kathrine Halsøy, Timofey Kondratiev, Torkjel Tveita, Lars J. Bjertnaes

**Affiliations:** ^1^Anesthesia and Critical Care Research Group, Faculty of Health Sciences, Department of Clinical Medicine, University of Tromsø, The Arctic University of Norway, Tromsø, Norway

**Keywords:** constant flow perfusion, constant pressure perfusion, fluid filtration coefficient, hypothermia, isolated rat lungs, lung edema, BAL/plasma protein concentration ratio

## Abstract

**Background:**

Victims of severe accidental hypothermia are prone to fluid extravasation but rarely develop lung edema. We hypothesize that combined hypothermia-induced increase in pulmonary vascular resistance (PVR) and a concomitant fall in cardiac output protect the lungs against edema development. Our aim was to explore in hypothermic-isolated blood-perfused rat lungs whether perfusion at constant pressure influences fluid filtration differently from perfusion at constant flow.

**Methods:**

Isolated blood-perfused rat lungs were hanging freely in a weight transducer for measuring weight changes (ΔW). Fluid filtration coefficient (Kfc), was determined by transiently elevating left atrial pressure (Pla) by 5.8 mmHg two times each during normothermia (37°C) and during hypothermia (15°C). The lung preparations were randomized to two groups. One group was perfused with constant flow (Constant flow group) and the other group with constant pulmonary artery pressure (Constant PPA group). Microvascular pressure (Pmv) was determined before and during elevation of Pla (ΔPmv) by means of the double occlusion technique. Kfc was calculated with the formula Kfc = ΔW/ΔPmv/min. All Kfc values were normalized to predicted lung weight (P_LW_), which was based on body weight (BW) according to the formula: P_LW_ = 0.0053 BW − 0.48 and presented as Kfc_PLW_ in mg/min/mmHg/g. At cessation, bronchoalveolar lavage (BAL) fluid/perfusate protein concentration (B/P) ratio was determined photometrically. Data were analyzed with parametric or non-parametric tests as appropriate. *p* < 0.05 considered as significant.

**Results:**

Perfusate flow remained constant in the Constant flow group, but was more than halved during hypothermia in the Constant PPA group concomitant with a more fold increase in PVR. In the Constant flow group, Kfc_PLW_ and B/P ratio increased significantly by more than 10-fold during hypothermia concerted by visible signs of edema in the trachea. Hemoglobin and hematocrit increased within the Constant flow group and between the groups at cessation of the experiments.

**Conclusion:**

In hypothermic rat lungs perfused at constant flow, fluid filtration coefficient per gram P_LW_ and B/P ratio increased more than 10-fold concerted by increased hemoconcentration, but the changes were less in hypothermic lungs perfused at constant PPA.

## Introduction

In human, accidental hypothermia is defined as an unintentional lowering of the core temperature to below 35°C following exposure to cold. Traditionally, the condition has been characterized by different stages of severity based on the prevailing body temperature, as mild hypothermia (32–35°C), moderate hypothermia (28–32°C), and severe hypothermia (< 28°C) (1–4).

Severe accidental hypothermia is associated with a marked decrease in cardiac output and systemic arterial pressure concerted by increases in systemic vascular resistance and pulmonary vascular resistance (PVR). Previous researchers have noticed that in dogs, when cooled to a core temperature of 17°C, cardiac output decreases exponentially to only 10% of its value at normal core temperature (5, 6). Correspondingly, Tveita and his coworkers noticed a fall in cardiac output to approximately 13% of its value during normothermia when lowering temperature in the aortic arc of rats to 13–15°C (7).

Investigators recently reported that induction of hypothermia causes extravasation of water and proteins independent of whether the condition is due to surface cooling or core cooling. Fluids and proteins shifting from the intravascular to the interstitial compartment resulted in edema in most organs, but not in the lungs (8).

The prognosis of victims of accidental hypothermia depends on the circumstances causing the cooling and the cardiorespiratory condition of the patient from the scene of accident to the center where adequate resuscitation can take place (9). The risk of respiratory and circulatory arrest increases with a decrease in core temperature to below 28°C, and estimated mortality rate varies between 30 and 80% (10).

Between 1985 and 2012, the University Hospital of North Norway attempted to rewarm totally 34 patients with hypothermic cardiac arrest on cardiopulmonary bypass (CPB). Nine patients survived. Notably, three of the survivors needed supplemental extracorporeal membrane oxygenation (ECMO) because their conditions complicated with acute respiratory distress syndrome (ARDS) and/or cardiopulmonary instability after the rewarming (9). One of the victims, a female skier, arrived to the hospital with cardiac arrest of nearly 3 h duration and a core temperature of 13.7°C. Post rewarming, her condition deranged with cardiorespiratory failure necessitating 5 days of ECMO support. Two years after the accident, she had resumed all her previous activities and displayed no sequelae (11).

A so-called “rewarming shock” might occur upon rewarming after hypothermia. This is believed to be associated with myocardial intracellular Ca^2+^ overload, which promotes backward failure and subsequently leads to increased microvascular pressure and emergence of lung edema (12). Moreover, activation of leukocytes and various inflammatory mediators may damage endothelial integrity and indirectly increase pulmonary microvascular permeability, thus, causing fluid accumulation and derangement of gas exchange (13, 14). Besides these direct effects of hypothermia, CPB *per se* has been shown to increase the extravascular lung water content by as much as 35–40%, in comparison with the preoperative values. Furthermore, after CPB investigators have reported bronchoalveolar lavage (BAL)/plasma protein concentration ratios of between 4 and 20 times above normal (14–16). Although our knowledge about the pathophysiology and the treatment of accidental hypothermia have progressed (11), several aspects need further elucidation.

Whether it is the fall in body temperature or the rewarming *per se*, or both combined, that is responsible for the cardiorespiratory failure in survivors of hypothermic cardiac arrest, remains unknown. However, we should bear in mind the possibility that both cardiac dysfunction, anesthesia and the type of extracorporeal circulation *per se*, as well as the hemodynamic and metabolic changes, all might play a role (12, 14–16).

We hypothesize that lung fluid extravasation during severe hypothermia may develop as a combined result of cardiac failure and cold-induced increase in microvascular permeability. However, possibly a clinically detectable lung edema could be masked by hypothermia-induced increase in PVR concomitant with a decrease in cardiac output. To test this hypothesis, we compared the fluid filtration coefficient and the BAL/perfusate protein concentration ratio (16) in isolated rat lungs that were perfused with autologuous rat blood at constant flow or at constant pulmonary artery pressure, respectively, during subsequent exposures to normothermia and hypothermia.

## Materials and Methods

Sprague-Dawley female rats weighing 250–350 g were acclimatized to the laboratory environment with free access to food and water for 1 week before the experiments.

The Animal Care Committee of the University of Tromsø under the Norwegian National Animal Research Authority approved the study (FOTS id 4590/Project number 29/12).

### Anesthesia

The rats were anesthetized with a combination of fentanyl and fluanisone (Hypnorm^®^, Janssen Pharmaceutica, Beerse, Belgium) and midazolam (Dormicum^®^, F Hoffman-La Roche AG, Basel, Switzerland) at a dose of 0.01–0.05 mg per 100 g and 1.0–1.75 mg per 100 g, respectively.

### Lung Isolation

Rats were tracheostomized with a 2 mm inner diameter stainless steel cannula and subsequently ventilated with air with a volume-controlled small-animal ventilator (NEMI, Medway) at 70 inflations/min, tidal volumes (V_T_) of 2–3 ml, and positive end-expiratory pressure of 2.0 cm H_2_O, as described previously from our group (17). In short, following a median sternotomy, we freed the lungs and the main vessels of adjacent tissue and cut and ligated esophagus. We placed loose ligatures around the inferior vena cava above the diaphragm and around the pulmonary artery, the aorta, and the superior vena cava above the heart level. Heparin 250 IU dissolved in saline was injected slowly into the left ventricle whereupon the ligature around the inferior vena cava was tightened, ventilation stopped, and the heart–lung preparation was removed from the chest and placed on parafilm. A stainless steel cannula of inner diameter 2.5 mm with two T-shaped outlets, one for monitoring pulmonary artery inflow pressure (PPA) and another serving as an air trap, were inserted into the pulmonary artery and the preplaced ligature was tightened. Correspondingly, a 4-mm inner diameter stainless steel outflow cannula with a T-shaped outlet for registration of left atrial pressure (Pla) was inserted *via* the left ventricle into the left atrium. The cannula was fixed with a cotton band around the heart. Then, the lungs together with the cannulas were moved to a thermostated (37°C) humidified perspex chamber, where the lung preparation was hanging freely suspended by the air-trap tubing of the inflow cannula to the hook of a weight transducer (FT 30°C, Grass Instruments, Quincy, MA, USA). This allowed continuous registration of weight changes that we sampled together with other recorded variables into the data acquisition program LabView (National Instrument Corporation, Austin, TX, USA). All the cannulas were manufactured by the Mechanical Workshop, Uit, The Arctic University of Norway, 9037 Tromsø, Norway.

### Blood Donors

In each experiment, two blood donor rats were anesthetized with the same combination of anesthetics as described above. The chest was opened and the rat was placed in Trendelenburg’s position. Following direct visual puncture of the right ventricle, blood was drawn into a 10 ml syringe, which was prepared with Heparin Sodium (1,000 IU/ml), 400 IU (Wockhardt UK Ltd., Wraxham, UK) without conservatives.

### Lung Perfusion

The pulmonary artery was perfused by means of a roller pump (Masterflex L/S, mod. 7523-70, Cole-Parmer Instrument Co., IL, USA). At the start of the experiment, air was evacuated *via* the air-trap tubing by flushing the perfusion system with Krebs–Ringer’s solution, which was bubbled trough with room air containing 5% CO_2_. The Krebs–Ringer’s solution was subsequently replaced by autologous rat blood. The blood perfusate was pumped into the pulmonary artery from a thermostated reservoir and re-circulated *via* the cannula in the left atrium. We aimed at keeping constant perfusate flow (*Q*, milliliters per minute) of approximately 15 ml/min or constant pulmonary artery pressure of 15 mmHg at baseline.

### Lung Ventilation and Temperature Control

The lungs were ventilated with a Harvard rodent ventilator (Model 683, Holliston, MA, USA) at a tidal volume (V_T_) of 2 ml of a gas mixture containing 21% O_2_, 5% CO_2_, and 74% N_2_ at a respiratory rate of 70 breaths/min and positive end-expiratory pressure of 2 cm H_2_O. Airway peak inspiratory pressure (not presented) was recorded in the trachea with a pressure transducer (Transpac III; Abbott, North Chicago, IL, USA).

The temperatures in the blood reservoir and in the chamber were constantly recorded (not presented). Perfusate samples were analyzed for blood gases at baseline and at the end of the experiment (not presented). Total lung weight including cannulas, were measured at baseline and at the end of experiment, before BAL was performed.

### Measurements and Calculations

PPA and Pla were measured with pressure transducers (Transpac III; Abbott, North Chicago, IL, USA). *Q* was determined by sampling blood perfusate from the left atrial cannula in a graded 20 ml tube for 1 min at the end of experiment. A ladder-like tube allowed Pla to be raised intermittently by 5.8 mmHg for 6 min during perfusion by clamping the lowest tube of the “ladder,” first two times with 30 min intervals during normothermia, each time preceded by baseline measurements. After approximately 30 min of cooling to a perfusate temperature of 15°C, the baseline measurements and the Pla-elevations each were repeated two times during hypothermia. PVR was determined as (PPA − Pla)/*Q*.

Pulmonary microvascular pressure (Pmv) was determined by means of the double occlusion method. The pulmonary artery tubing was occluded distal to the pulmonary artery pressure transducer, and simultaneously the outflow was occluded downstream of the left arterial pressure transducer and the roller pump was stopped. With zero flow in the system, the pressure in the lung vasculature stabilized rapidly and we determined Pmv at baseline and with a Pla-elevation of 5.8 mmHg. The increase in pulmonary microvascular pressure (ΔPmv) was calculated as the difference between Pmv during elevation of Pla and Pmv at baseline.

The weight transducer was checked for linearity by hanging 0.5 and 1 g weights on the hook before every experiment and the procedure was repeated during the experiment by hanging a 0.5-g weight at the point of insertion of the pulmonary artery cannula into the heart. The weight gain (ΔW) was displayed and the lung fluid filtration coefficient was calculated from the formula Kfc = ΔW/ΔPmv/min, as demonstrated by other researchers from our laboratory. Weight changes were measured with the weight transducer during all the four Pla elevations. After determining Kfc, all values were normalized to predicted lung weight (P_LW_). P_LW_ was calculated based on the body weight (B_W_) as: P_LW_ = 0.0053 B_W_ − 0.48 g, and Kfc_PLW_ was presented in mg/min/mmHg/g (17).

### Experimental Protocol

We randomized the isolated rat lung preparations into two groups. One group was perfused at constant flow (Constant flow group, *n* = 7). The other group was perfused at constant pulmonary artery pressure (Constant PPA group, *n* = 7). Both groups were exposed to two Pla (Pla1 and Pla2) elevations during normothermia subsequently followed by lowering the temperatures of perfusate and perfusion chamber to 15°C, whereupon the Pla elevations were repeated two times during hypothermia (Pla3 and Pla4), each time preceded by baseline measurements (BL1-BL4). All lung preparations were weighed with all cannulas in place before the start of perfusion and at cessation of the experiment. In the Constant flow group, the lungs were perfused with the same flow rate during both the normothermic and the hypothmeric period. In this group, three lung preparations were discarded after three Pla-elevations because of excessive edema formation and weight increases beyond the calibrated range of the weight transducer. This made quantitation of weight changes impossible and increased the risk of exposing the ventilator to edema fluid. In the Constant PPA group, we aimed at keeping PPA constant by gradually reducing perfusate flow (*Q*) throughout the cooling period. In that group, one lung pair was discarded because of a technical problem. All the discarded lung experiments were replaced. Fourteen out of a total of 18 lung preparations completed the experiments, thus the drop-out rate was 4/18. Double occlusion for determination of Pmv was performed at the first baseline (BL1) and the first Pla elevation (Pla1), respectively, and repeated at the end of experiment in both groups (Pla4).

### BAL/Perfusate Protein Concentration Ratio

Trachea was cut and a 16 G cannula was inserted into the right bronchus. The cannula was ligated and 3 ml of a phosphate buffer was instilled slowly (16). After approximately 10 s, the bronchoalveolar lavage (BAL) fluid was withdrawn into the syringe and analyzed with the Bradford protein assay. Simultaneously, a perfusate sample was centrifuged and subsequently analyzed for protein concentration using the same assay.

### Data Analysis

Data were stored and calculated in MS Excel (MS Office Professional 2010, Microsoft Corporation, USA). The SigmaPlot software (SigmaPlot ver. 13, Systat Software Inc., Chicago, IL, USA) was used for statistical analyses and drawing the figures. Data distribution was assessed using Shapiro–Wilk test. Normally distributed data were assessed by Student’s *t*-test or repeated measures analysis of variance (RM ANOVA) for intergroup and intragroup differences, respectively. If normal distribution could not be demonstrated, Mann–Whitney Rank Sum Test s or RM ANOVA on Ranks was used to assess intergroup and intragroup differences, respectively. If the *F* value was greater than critical, RM ANOVA was followed by Dunnett’s *post hoc test* for intragroup comparisons. The results were presented as mean ± SD for normally distributed data and as median with interquartile range as vertical boxes and 10^th^ and 90^th^ percentile error bars for non-normally distributed data. *p* < 0.05 was regarded as statistically significant.

## Results

### Hemodynamics

**Figure 1:**
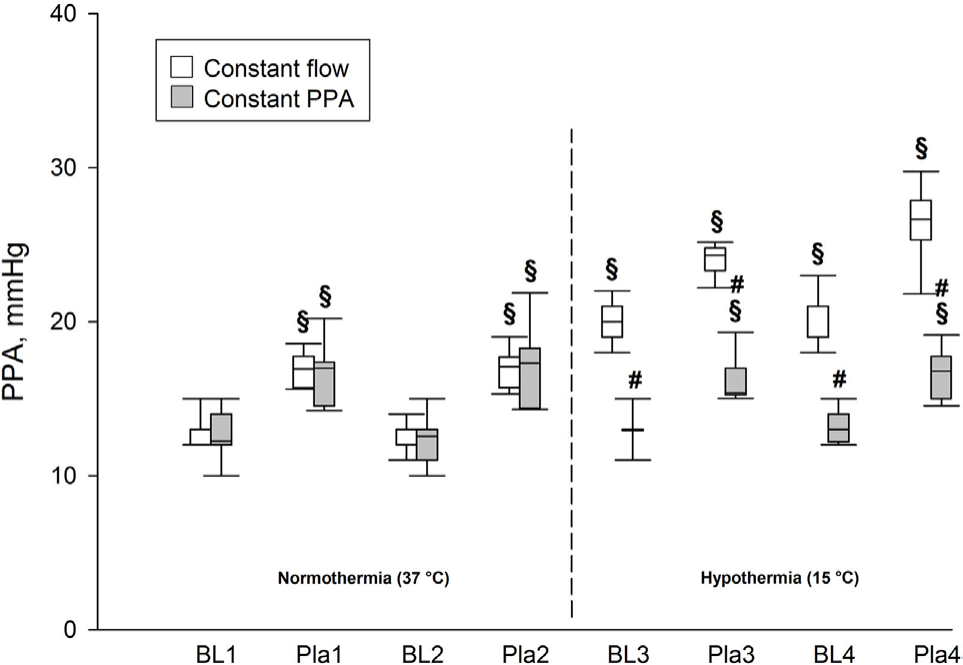
**Pulmonary artery pressure in isolated blood-perfused rat lungs**. PPA, pulmonary artery pressure; data presented as vertical boxes with median and interquartile range with 10th and 90th percentile error bars; BL1–BL4, baseline measurements; Pla1–Pla4, left atrial pressure elevations by 5.8 mmHg; vertical dashed line, boarder between normothermia and hypothermia; ^§^*p* < 0.05 compared with intragroup baseline (BL1); ^#^*p* < 0.05 compared with Constant flow group.

**Figure 2:**
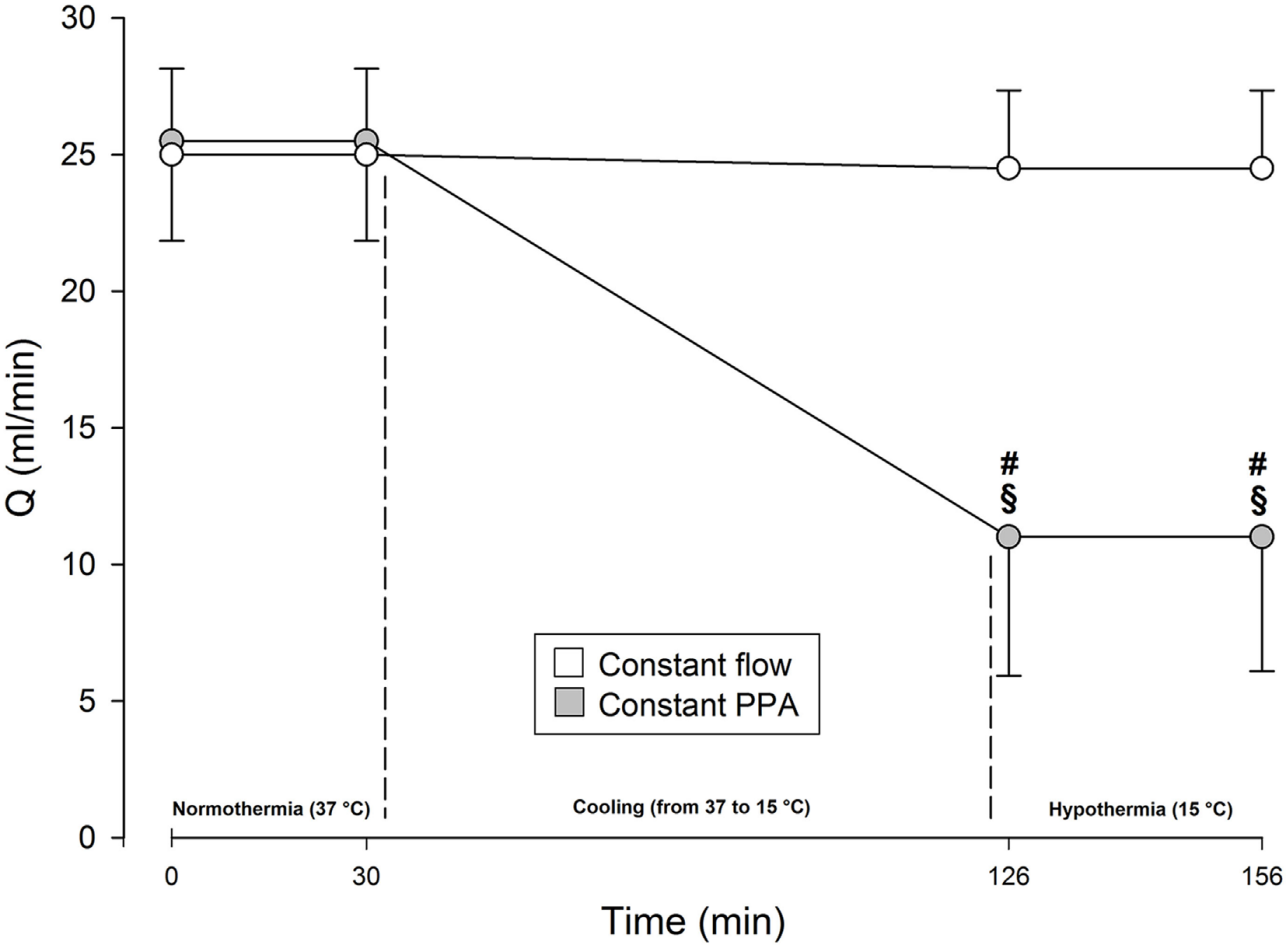
**Perfusate flow in isolated blood-perfused rat lungs**. *Q*, perfusate flow, milliliters per minute; Constant flow group, perfused normothermic and hypothermic with constant flow; Constant PPA group, perfused normothermic and hypothermic with constant pulmonary artery pressure; data presented as mean ± SD; ^§^*p* < 0.05 compared with intragroup baseline; ^#^*p* < 0.05 compared with Constant flow group.

**Figure 3:**
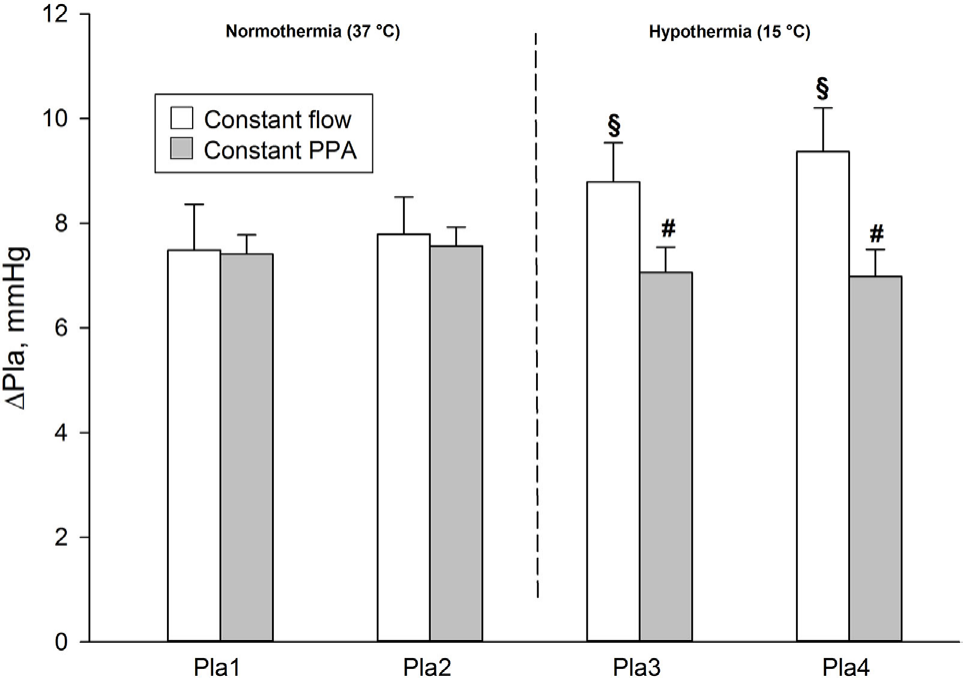
**Measured elevations of left atrial pressure in isolated blood-perfused rat lungs**. ΔPla, measured after elevations of left atrial pressure by 5.8 mmHg. Data presented as mean ± SD; ^§^*p* < 0.05 compared to intragroup baseline; ^#^*p* < 0.05 compared with Constant flow group.

**Figure 4:**
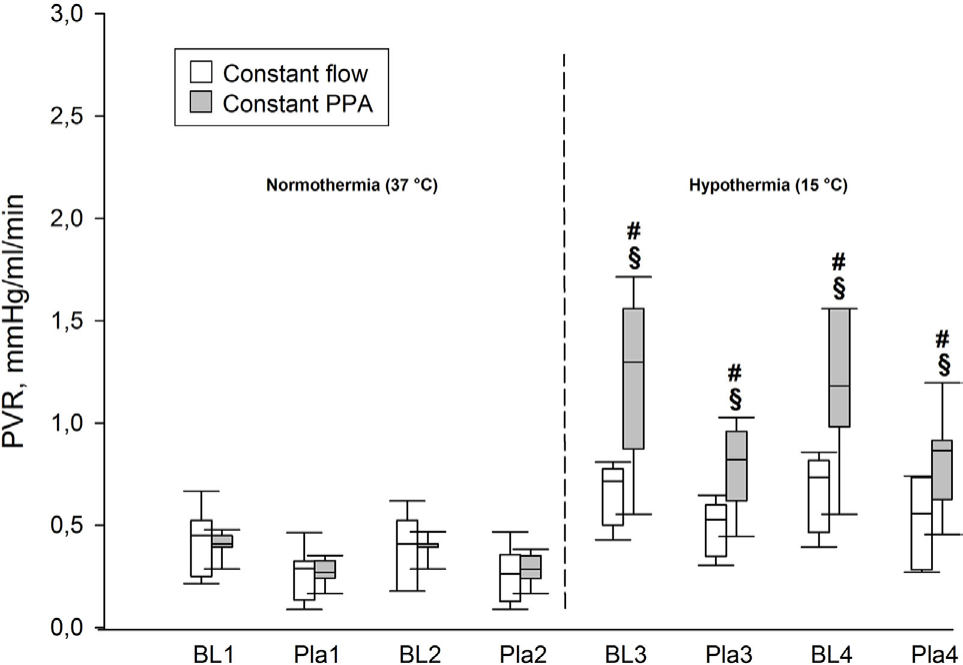
**Pulmonary hemodynamics**. PVR, pulmonary vascular resistance, mmHg/ml/min; data presented as vertical boxes with median and interquartile range with 10th and 90th percentile error bars; ^§^*p* < 0.05 compared with intragroup baseline; ^#^*p* < 0.05 compared with Constant flow group.

**Figure 5:**
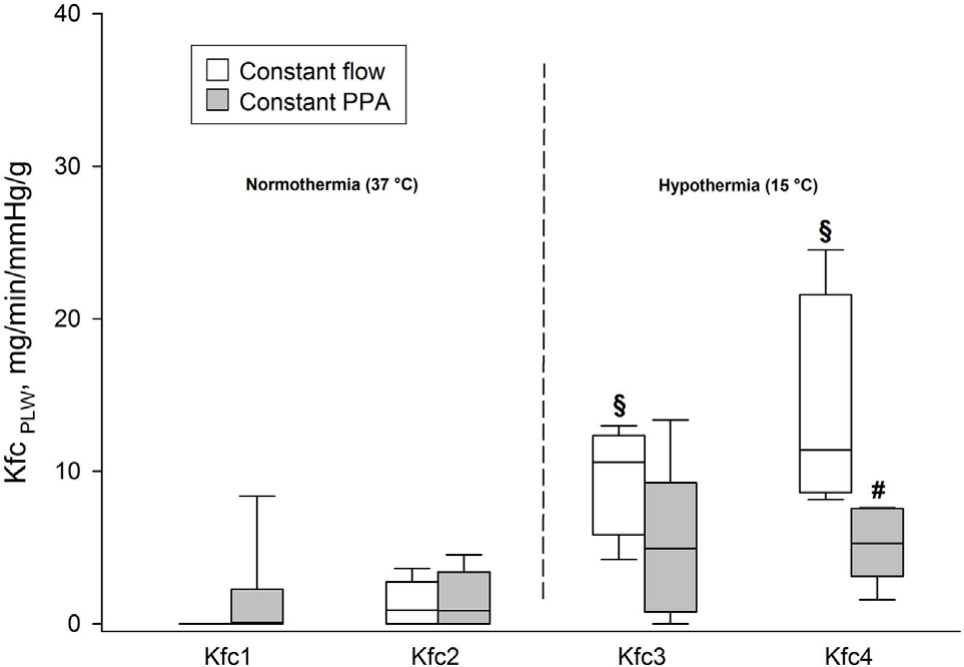
**Fluid filtration coefficient**. Kfc_PLW_, fluid filtration coefficient calculated on the basis of predicted lung weight, ml/min/mmHg/g; data presented as vertical boxes with median and interquartile range with 10th and 90th percentile error bars; ^§^*p* < 0.05 compared with intragroup baseline; ^#^*p* < 0.05 compared with Constant flow group.

During normothermia, we found no significant intergroup differences in PPA, *Q*, Pla or PVR, neither at baseline (BL1 and BL2) nor at the elevations of Pla (Pla1 and Pla2) as depicted in Figures 1–5. As shown in Figure 1, in both groups PPA increased compared to intragroup baseline during Pla1 and Pla2 (*p* < 0.05), but with no intergroup difference. In the Constant PPA group, PPA remained lower during hypothermia in comparison with the Constant flow group (*p* < 0.05). As displayed in Figure 2, *Q* decreased markedly during hypothermia in the Constant PPA group in comparison with the Constant flow group and with intragroup baseline values during normothermia (*p* < 0.05). In the Constant flow group, the measured rises in Pla (ΔPla), after cooling, were significantly higher as compared with intragroup baseline and the Constant PPA group (Figure 3), but ΔPmv did not change significantly within or between groups (not shown). Hypothermia prompted a marked rise in PVR (Figure 4) in the Constant PPA group, both in comparison with intragroup baseline and the Constant flow group (*p* < 0.05).

### Kfc_PLW_

We found no significant differences in Kfc_PLW_ within or between the groups during the first two elevations of Pla determined under normothermic conditions (*p* < 0.05). In the Constant flow group, Kfc_PLW_ increased in comparison both with intragroup baseline and with the Constant PPA group at Pla4 (*p* < 0.05), as shown in Figure 5.

### B/P Protein Concentration Ratio

**Figure 6:**
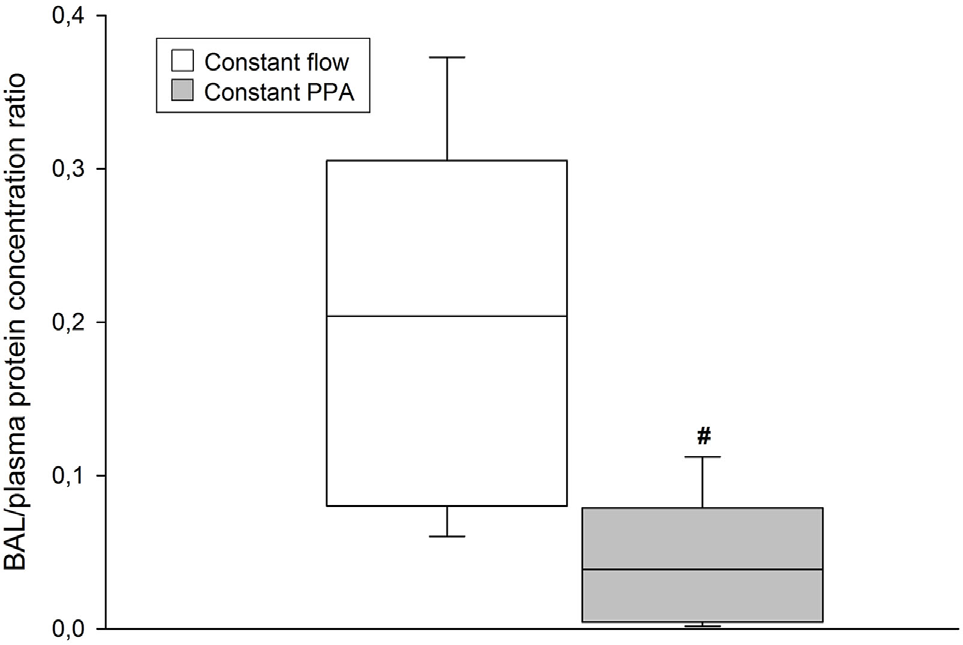
**BAL/plasma protein concentration**. BAL, bronchoalveolar lavage; data presented as vertical boxes with median and interquartile range with 10th and 90th percentile error bars at cessation of the experiment; ^#^*p* < 0.05 between Constant flow group and Constant PPA group.

The Constant PPA group presented with no clinical signs of edema. In contrast, the Constant flow group displayed edema fluids in the trachea of all lungs. As depicted in Figure 6, B/P protein concentration ratio increased significantly in the Constant flow group, in comparison with in the Constant PPA group (*p* < 0.05).

In the Constant flow group, four preparations could not be perfused beyond the third Pla elevation because pink foam occurred in airways and ventilator tubes. These experiments were excluded from the study, thus leaving seven lung pairs in each group for comparison.

### Lung Weight and Hematological Variables

Table 1 shows a 40% intragroup increase in total lung weight in Constant flow group as compared with 11% in Constant PPA group and a significant intergroup difference. Hemoglobin also increased by approximately 30% from baseline at cessation of the experiment in the Constant flow group, and correspondingly, by only 5% in the Constant PPA group constituting significant intragroup and intergroup differences (*p* < 0.05). Hematocrit also increased significantly within and between the groups (*p* < 0.05) as listed in Table 1.

**Table 1:** **Hematology and lung weight**.

Variable	Time (min)			

	Pla1 (0)	Pla2 (30)	Pla3 (126)	Pla4 (156)
**Hb (g/dL)**				
Constant flow group	9.9 ± 0.9	–	–	12.9 ± 1.6^§^
Constant PPA group	9.9 ± 1.5	–	–	10.4 ± 1.8*
**Hct (%)**				
Constant flow group	30.7 ± 2.7	–	–	39.9 ± 4.3^§^
Constant PPA group	30.6 ± 4.5	–	–	31.6 ± 6.6*
**Total lung weight (g)**				
Constant flow group	13.1 ± 0.3	–	–	18.6 ± 3.1
Constant PPA group	13.3 ± 0.3	–	–	15.0 ± 0.3*

*Pla1–Pla4, left atrial pressure elevations by 5.8 mmHg; Time (min), time at start of Pla elevation; Hb, hemoglobin; Hct, hematocrit*.

*Data presented as the mean ± SD*.

*^§^p < 0.05 compared with intragroup baseline*.

*p < 0.05 compared with Constant flow group.

## Discussion

The present study demonstrates that under severe hypothermic conditions, isolated rat lungs perfused with blood at constant flow display significant increments in fluid filtration coefficient, lung weight, and BAL/plasma protein concentration ratio in concert with increased hemoconcentration, as compared with lungs perfused at constant pulmonary artery pressure. In the latter group, perfusate flow was more than halved in the presence of nearly 150% increase in PVR, but with no significant increase in hemoconcentration at cessation of the experiments. These findings suggest that a derangement of barrier functions occurs during severe hypothermia in lungs perfused at constant flow. However, apparently, these changes are less pronounced or masked in lungs perfused at constant pulmonary artery pressure.

Our findings support the notion that a loss of microvascular endothelial barrier function takes place in lungs exposed to severe hypothermia (18, 19). According to recent investigations, hypothermia promotes fluid shifts from the intravascular to the interstitial space (8, 20–24). Most of these observations originate from clinical and experimental studies involving the use of CPB. A widely held opinion is that extracorporeal circulation *per se* can promote lung inflammation and increase microvascular permeability (25). Interestingly, Hammersborg and coworkers studied surface-cooled pigs without the use of CPB and found that extravasation was most extensive in skin, muscle, and gastrointestinal tract whereas no spurs of edema appeared in the lungs (8). These findings are consistent with observations made in victims of severe accidental hypothermia. Most of those admitted to hospital with hypothermic cardiac arrest had clinically “dry lungs” upon arrival. However, lung edema emerged in some of them after they had been re-warmed and weaned off CPB (9, 26). It is close at mind to compare the initial absence of lung edema of these patients with that observed in the Constant PPA group of the present study.

When connected to a bypass between the right atrium and the aorta, lung blood flow is low until the heart resumes its pumping activity. Initially, reperfusion takes place against an increased PVR (8, 21, 27). Following cooling of pigs to rectal temperatures of 26–28°C, the investigators observed a drop in cardiac index to approximately one-third of that determined at normal rectal temperature and, simultaneously, a threefold increase in PVR, whereas pulmonary artery pressure remained unchanged. The lack of lung water accumulation in the study of Hammersborg et al. (8) is consistent with our observation of decreased fluid filtration concomitant with a sizeable decrease in perfusate flow in lungs perfused at constant PPA.

Previous investigators reported from experiments on isolated rat lungs, that PVR increased by 1–2% per degree decrease in perfusate temperature, but the etiology of this rise is not fully clarified. The authors suggest that the vascular walls become stiffer during cooling, and blood viscosity increases partly because of an approximately 2% increase in hematocrit for every 1°C decline in body temperature (20, 27, 28). In the present study, particularly the Constant flow group presented with significant increases in hemoglobin and hematocrit concentrations, in parallel with increased fluid filtration. According to Poiseulle’s law, the increase in viscosity might have contributed to the enhanced PVR in these experiments. The changes from baseline in hemoglobin and hematocrit concentrations were least in the Constant PPA group, consistent with the observation that outward filtration in that group was less as compared to Constant flow group. We suggest, as a possible explanation, that the lower fluid filtration in the Constant PPA group could be secondary to vasoconstriction and shrinking of the filtration area when exposed to hypothermia. However, we can neither strengthen nor discard this hypothesis since we neither measured vascular dimensions nor regional blood flow in these experiments.

In fact, blood vessels are elastic structures that exert retractive forces. This implies that when perfused with non-pulsatile flow at constant pressure, lung vessels tend to close in the presence of a vasoconstrictor stimulus, such as hypothermia (27). More than three decades ago, closure of lung vasculature was suggested as an explanation of the zero blood flow noticed during hypoxia-induced vasoconstriction in isolated rat lungs perfused at constant pulmonary artery pressure of up to 30 mmHg (29). Based on these observations, we suggest that the hypothermia-induced fall in cardiac output in victims of severe hypothermia might have prompted a de-recruitment (“critical closure”) in parts of the lung circulation, thereby reducing the area available for fluid filtration. In hypothermic individuals, we speculate that such vascular de-recruitment might persist until the microvascular transmural pressure has increased in response to regained pumping of the heart after weaning off CPB. The resulting increase in transvascular pressure might facilitate microvascular leaks.

In the Constant flow group, vascular de-recruitment was counteracted by a pump-driven increase in pulmonary artery pressure up to the level required to overcome the increased PVR. In the presence of deranged vascular endothelial barriers, the increments in both filtration area and pressure increase fluid filtration. The changes in Pmv did not reach statistically significant differences between the groups, but we admit that the increase in measured Pla was higher in the Constant flow group, as compared with the other group. This might have prevented de-recruitment and contributed to the increased fluid filtration in that group.

It is pertinent to ask what causes the hypothermia-induced increase in microvascular permeability, as evidenced by the increased Kfc_PLW_ and the BAL/plasma protein concentration ratio. We found no investigation on intact animals or in isolated lungs that has addressed this particular question. However, Diestel and coworkers studied human umbilical vein endothelial cells that were pre-treated with methylprednisolone and/or tacrolimus, incubated within a specially designed bioreactor or monolayers and that they exposed to deep hypothermia (17°C) followed by rewarming. When exposed to hypothermia, the endothelial cells changed into a more elongated shape with intercellular gap formation and increased permeability of endothelial monolayers. Opening and closing of the endothelial gaps were regulated with expression of extracellular signal proteins. The changes recovered upon rewarming and were inhibited in cells pre-treated with methylprednisolone and tacrolimus (19). Thus, a future investigation in isolated rat lungs should take advantage of these findings before testing the combination in a large animal model of severe accidental hypothermia. Moreover, other permeability-regulating proteins should be considered for a role in protecting the integrity of the alveolocapillary barrier as well.

We admit that the present study has several limitations. For the first, we lacked a normothermic control group. However, previous studies from our laboratory have shown that Pla increments of 5.8 mmHg can be repeated five times without significant increase in fluid filtration under normothermic conditions (17).

We should exercise great caution when extrapolating evidence obtained in isolated lungs to victims of severe accidental hypothermia. However, the rise in PVR, which was most prominent in the Constant PPA group, is consistent with previous observations made in various species including rats, pigs, and dogs (8, 17, 21, 30). At variation from isolated lungs that are perfused at constant flow or constant pressure, intact organisms adjust perfusion to the demands of the local tissues in an interaction between peripheral sensors and the central nervous system (31). With reports of cardiac output in animals as low as 10% of the normal at a core temperature of 17°C, we assume that larger parts of the circulation have been shut down although the pulmonary artery pressure tended to be maintained almost at the normal level during hypothermia in these animals (5, 6). Isolated lungs have no autonomic nerves or hormonal influences. However, despite lung circulation is locally regulated, the markedly reduced blood flow in response to hypothermia in lungs of the Constant PPA group, reminds highly on the changes taking place in hypothermic intact animals and even in man (5, 6, 8, 11, 32).

A combination of maintaining a relatively high perfusate flow (Figure 2), in addition to derangement of membrane integrity because of severe hypothermia, contributed to unconcealed edema formation in the Constant flow group. Occurrence of pink foamy liquid in trachea and ventilator tube before termination of the fourth Pla-elevation in three experiments was associated with a large increase in lung weight, which exceeded the calibration range of the weight transducer and made quantification of weight gain impossible. This was the reason for discarding these experiments in the Constant flow group. An additional reason was to avoid contaminating the ventilator with edema fluid. In contrast, in the Constant PPA group, all lung preparations fulfilled the whole experimental protocol except for one, which was discarded and replaced after dislocation of left atrial cannula during the normothermic period of the perfusion.

The way fluid filtration was determined also can be criticized. Precautions were taken by calibrating the weight both before and during the experiments. Even small alterations in the positioning of the lungs could affect the weight, and since the rat lungs with their connected cannulas and tubes were hanging in a weight transducer, the lungs made up only a small part of the total weight. After fulfillment of the first transient elevation of Pla and the concomitant weight increase had returned toward its basline value, the weight tended to decrease further to slightly below baseline in nearly half of the lungs. We believe that this initial weight loss was secondary to reabsorption of extravasated Krebs–Ringer’s solution after we replaced the perfusate with whole blood of a higher oncotic pressure. Elevation of Pla also might have caused microvascular recruitment and increased inward fluid filtration secondary to an expansion of the filtration area. Already before the second elevation of Pla, the lung fluid filtration curve became more stable since Kfc_PLW_ was more reproducible most likely because the oncotic pressure was more stabilized. Since perfusate flow was higher in the Constant flow group, this resulted in an increase in ΔPla, which might have increased the filtration area of that group despite microvascular pressure (ΔPmv) did not differ significantly. As a consequence of these weaknesses, further experiments are warranted to confirm our findings under technically more optimal conditions, including an automatic feedback loop varying perfusate flow while keeping pulmonary artery pressure constant in the Constant PPA group.

## Conclusion

During hypothermia, lung fluid filtration coefficient increased significantly in parallel with an approximately 10-fold increase in BAL/plasma protein concentration ratio and a raise in hemoglobin and hematocrit under constant flow perfusion. All the latter changes were less pronounced in lung preparations perfused at constant pulmonary artery pressure. Although caution must be taken when extrapolating results from experiments on isolated rat lungs to humans, we suggest that a parallel increase in PVR and decrease in pulmonary blood flow might prevent lung edema from developing in victims of severe accidental hypothermia.

## Author Contributions

All the investigators participated in the design of the study. KH and TK carried out the experiments and analyzed the data. TT administered the study and suggested improvements to the manuscript. KH and LB drafted the manuscript. All the authors read and approved the finished manuscript.

## Conflict of Interest Statement

The authors declare that they performed the present study in the absence of any commercial or financial relationships that could be interpreted as a potential conflict of interest.
